# Metformin use and respiratory outcomes in asthma-COPD overlap

**DOI:** 10.1186/s12931-021-01658-3

**Published:** 2021-02-26

**Authors:** Tianshi David Wu, Ashraf Fawzy, Gregory L. Kinney, Jessica Bon, Maniraj Neupane, Vickram Tejwani, Nadia N. Hansel, Robert A. Wise, Nirupama Putcha, Meredith C. McCormack

**Affiliations:** 1 Section of Pulmonary, Critical Care, and Sleep Medicine, Baylor College of Medicine, Houston, TX USA; 2 Center for Innovations in Quality, Effectiveness and Safety, Michael E. DeBakey VA Medical Center, Houston, TX USA; 3 Division of Pulmonary and Critical Care Medicine, Johns Hopkins School of Medicine, 1830 E. Monument St. 5th Floor, Baltimore, MD 21205 USA; 4 Department of Epidemiology, Colorado School of Public Health, Aurora, CO USA; 5 Division of Pulmonary, Allergy, and Critical Care Medicine, University of Pittsburgh, Pittsburgh, PA USA; 6 VA Pittsburgh Healthcare System, Pittsburgh, PA USA; 7 Department of Critical Care Medicine, National Institutes of Health, Bethesda, MD USA

**Keywords:** Asthma-COPD overlap, Exacerbations, Metformin

## Abstract

**Background:**

Metformin is associated with improved respiratory outcomes in asthma; however, metformin in COPD and asthma-COPD overlap (ACO) remains unexplored.

**Objective:**

To determine the association between metformin use and respiratory outcomes in COPD and ACO.

**Study design and methods:**

Participants with COPD (FEV1/FVC < 0.70) in the Genetic Epidemiology of COPD study (COPDGene®) were categorized as ACO (n = 510), defined as concurrent physician-diagnosed asthma before age 40 years, or COPD alone (n = 3459). We estimated the association of baseline metformin use with (1) rate of total and severe respiratory exacerbations during follow-up, (2) cross-sectional St. George’s Respiratory Questionnaire (SGRQ) score, six-minute walk distance (6MWD), and post-bronchodilator FEV1 percent predicted (FEV1pp), and (3) 5-year change in SGRQ, 6MWD, and FEV1pp. We also examined change in SGRQ, 6MWD and FEV1pp among participants who initiated metformin during follow-up (n = 108) compared to persistent metformin non-users (n = 2080). Analyses were adjusted for sociodemographic factors, medications, and comorbidities.

**Results:**

Among participants with ACO, metformin use was associated with lower rate of total (adjusted incidence rate ratio [aIRR] 0.3; 95% confidence interval [95%CI] 0.11, 0.77) and severe exacerbations (aIRR 0.29; 95%CI 0.10, 0.89). Among participants with COPD alone, there was no association between metformin use with total (aIRR 0.98; 95%CI 0.62, 1.5) or severe exacerbations (aIRR 1.3; 95% CI 0.68, 2.4) (p-interaction < 0.05). Metformin use was associated with lower baseline SGRQ score (adjusted mean difference [aMD] − 2.7; 95%CI − 5.3, − 0.2) overall. Metformin initiation was associated with improved SGRQ score (aMD –10.0; 95% CI − 18.7, − 1.2) among participants with ACO but not COPD alone (p-interaction < 0.05). There was no association between metformin use and 6MWD or FEV1pp in any comparison.

**Conclusions:**

Metformin use was associated with fewer respiratory exacerbations and improved quality of life among individuals with ACO but not COPD alone. Results suggest a potential role for metformin in ACO which requires further prospective study.

*Trial Registry:* NCT00608764

## Background

Individuals with asthma-COPD overlap (ACO) share clinical and physiologic features of both asthma and COPD [1]. While there is inconsistency regarding its diagnostic criteria and disagreement on whether it represents a distinct condition, it is clear that these individuals are especially vulnerable to poor health outcomes and experience greater morbidity than individuals with COPD alone [2, 3]. Because medication trials for asthma and COPD have generally excluded individuals with the opposing condition, evidence-based treatments for ACO remain sparse [4].

Metformin is an oral medication that is commonly indicated for the treatment of type 2 diabetes. In translational and epidemiologic studies of asthma, metformin has been associated with diminished allergic airway inflammation [5] and lower risk of disease exacerbation [6]. However, in studies of COPD, metformin did not reduce risk of hyperglycemia or risk of re-hospitalization for COPD [7]. Further, a study of Taiwan’s National Health Insurance Program reported a higher risk of pneumonia and hospitalization for COPD among metformin users [8]. These contrasting reports create uncertainty with respect to potential effects of metformin in both COPD and ACO.

Consequently, we sought to determine (1) the association of metformin use and respiratory outcomes among individuals with ACO and (2) whether these associations differ between ACO and COPD alone. We hypothesize that metformin use would be associated with lower risk of respiratory morbidity preferentially among individuals with ACO.

## Study design and methods

### Cohort description

The Genetic Epidemiology of COPD (COPDGene) study is an ongoing multi-center observational study with the goal of identifying genetic factors associated with COPD comprised of self-identified non-Hispanic whites or African-Americans aged 45–80 years with ≥ 10 pack-year smoking history. Full study details are reported elsewhere [9]. Participants underwent in-person characterization at baseline (phase 1) and year five (phase 2) in addition to telephone or web-based follow-ups at 3- to 6-month intervals. The current analysis includes follow-up data current as of July 31, 2018.

**Fig. 1:**
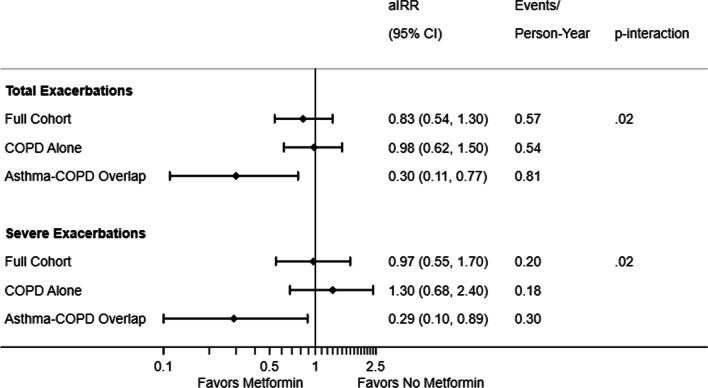
Incidence rate ratio (95% confidence interval) of metformin users compared to non-users stratified by presence of asthma diagnosed by a doctor before age 40. Models are adjusted for age, sex, race, education, post-bronchodilator FEV_1_ percent predicted, self-reported respiratory exacerbation in the prior 12 months (yes/no), smoking status, body mass index, inhaled corticosteroid use, oral corticosteroid use, other diabetes medication use, and comorbidity count. The p-value shown is for the interaction representing effect modification of Asthma-COPD overlap status on the association of metformin use with exacerbation rate

The analytic cohort was defined as participants with COPD, defined as FEV1/FVC < 0.70, who provided medication data at baseline and contributed follow-up data. Participants with ACO were defined as those with COPD and a diagnosis of asthma by a physician before the age of 40 years [10]. In sensitivity analyses, we also tested asthma diagnosed by a physician at any age and self-reported asthma not requiring a physician diagnosis.

### Exposure definitions

Metformin use was extracted from participant reported medication inventories obtained at baseline and 5-year follow-up (Additional file 1: Table S1). Medication tables were manually reviewed for misspellings and brand names of metformin to minimize misclassification. For the main analysis, the comparison was between participants who reported metformin use versus no metformin use at baseline.

We also investigated changes in respiratory outcomes associated with initiation of metformin. A metformin initiator was defined as a participant who reported no metformin use at baseline but metformin use at 5-year follow-up. In this analysis, the comparison was between initiators of metformin versus participants who reported no metformin use at baseline and follow-up.

### Outcome and covariate definitions

The primary outcome was the rate of total and severe respiratory exacerbations. Using automated telephone calls and web-based data entry, participants were asked to quantify the number of exacerbations and the outcome of each event at 3 to 6 month intervals. Severe exacerbations were flares that resulted in emergency department visit or hospitalization. Total exacerbations include severe exacerbations and exacerbations that resulted in an antibiotic or corticosteroid prescription.

Medication use and sociodemographic factors were assessed by questionnaire. Quality of life was assessed by the St. George Respiratory Questionnaire (SGRQ) score [11], which ranges from 0 to 100, with higher scores reflecting worse impact. The minimal clinically important difference (MCID) in asthma and COPD is 4 [12]. Functional ability was assessed by the 6-min walk distance (6MWD) which has a MCID of 90 feet in COPD [13]. Post-bronchodilator spirometry was performed in accordance with American Thoracic Society guidelines, and predicted values were calculated [14], from which post-bronchodilator FEV_1_ percent predicted (FEV_1_pp) was extracted.

### Analytic approach

The association of metformin use and rate of total and severe respiratory exacerbations was estimated by a negative binomial model with years of follow-up as an offset. Cross-sectional associations of metformin use with SGRQ, 6MWD, and FEV1pp at baseline were estimated by linear regression.

Differences in the change of SGRQ score, 6MWD, and FEV1pp associated with prevalent and incident metformin use between baseline and follow-up were estimated by linear mixed models, accounting for repeated measurements within participant using an unstructured covariance, with random intercept by center. The difference attributable to metformin was represented by a metformin-time interaction.

All models were adjusted for age, sex, race, education (less than high school, high school or greater), smoking status (current, former), body mass index (BMI; underweight, normal or overweight, obese), inhaled corticosteroid use, oral corticosteroid use, other diabetes medication use, FEV_1_pp, and a comorbidity count [15]. Exacerbation models and cross-sectional models of SGRQ, 6MWD and FEV1pp were additionally adjusted for respiratory exacerbation in the prior year (yes, no). FEV_1_pp was not included for estimates of FEV_1_pp as an outcome. In longitudinal models of prevalent metformin use, smoking status, BMI, and FEV_1_pp were included as time-varying covariates. Time-varying inhaled and oral corticosteroid use were also included in longitudinal models of incident metformin use.

Interaction by the presence or absence of ACO was tested and stratified estimates were produced. A two-sided p-value < 0.05 was accepted as statistically significant. All analyses were performed in SAS 9.4 (Cary, NC).

### Regulatory statement

COPDGene is performed with local IRB authorization from participating centers and is registered at ClinicalTrials.gov (NCT00608764). All study participants provided written informed consent.

## Results

### Cohort description

From the initial cohort, 3969 individuals with COPD who had complete baseline medication data and accrued follow-up time were identified (Additional file 1: Figure S1). Mean (SD) participant age was 63.6 (8.5) and 45% identified as female (Table 1). Participants who used metformin were older, had a greater number of comorbidities and lower proportion of females, and were less likely to be currently smoking. Of this group, 510 (13%) reported a diagnosis of asthma made by a physician before the age of 40 and were thus classified as ACO. On average, participants with ACO were younger, more commonly female, black, or obese, with more severe obstruction on spirometry, and more comorbidities. (Additional file 1: Table S1). There was no difference in the prevalence of ACO between metformin users and non-users.

**Table 1:** Baseline characteristics of included participants by baseline metformin use (n = 3969)

Characteristic [N (%) or Mean ± SD]	Metformin Non-Users (n = 3728)	Metformin Users (n = 241)	p-value
Age	63.4 ± 8.5	65.7 ± 7.5	< 0.01
Female gender	1703 (46)	82 (34)	< 0.01
Black race	747 (20)	43 (18)	0.4
GOLD stage			
GOLD 1	650 (17)	34 (14)	
GOLD 2	1602 (43)	107 (44)	0.2
GOLD 3	980 (26)	75 (31)	
GOLD 4	496 (13)	25 (10)	
Post-bronchodilator FEV1pp	57.3 ± 22.7	56.5 ± 20.7	0.6
Current smoker	1513 (41)	72 (30)	< 0.01
Body mass index			
Underweight	103 (3)	1 (1)	
Normal/overweight	2508 (67)	94 (39)	< 0.01
Obese	1117 (30)	146 (61)	
Exacerbation in prior year	1266 (34)	97 (40)	0.05
Physician-diagnosed asthma	818 (22)	59 (25)	0.4
Before age 40	471 (13)	39 (16)	0.1
Medications			
Inhaled corticosteroids	390 (11)	27 (12)	0.7
Oral corticosteroids	165 (5)	10 (4)	0.9
Insulin	18 (1)	5 (2)	0.01
Other diabetes medication	83 (2)	103 (43)	< 0.01
Comorbidities			
Comorbidity count	2.3 ± 1.7	4.1 ± 1.7	< 0.01
Coronary artery disease	327 (9)	48 (20)	< 0.01
Congestive heart failure	153 (4)	18 (8)	0.01
Stroke	125 (3)	10 (4)	0.5
Hypertension	1735 (47)	179 (74)	< 0.01
High cholesterol	1522 (41)	171 (71)	< 0.01

*GOLD* Global Initiative for Chronic Obstructive Lung Disease, *FEV1pp* forced expired volume in one second, percent predicted

### Prevalent metformin use and longitudinal respiratory exacerbations

Over 25,448 person-years, 14,512 total and 5032 severe exacerbations were recorded with a mean (SD) total exacerbation rate of 0.64 (1.1) and severe exacerbation rate of 0.24 (0.62). Participants with ACO had higher exacerbation rates compared to those with COPD alone. Metformin users were followed for an average of 6.5 ± 2.5 years (median 7.3 years, maximum 10.2 years), which was similar to the average 6.4 ± 2.7 years (median 7.4 years, maximum 10.4 years) among metformin non-users (p-value 0.5). Overall, there was no association of metformin use and total exacerbations (adjusted incidence rate ratio [aIRR] 0.83; 95% CI 0.54, 1.3) or severe exacerbations (aIRR 0.97; 95% CI 0.55, 1.7). However, among participants with ACO, metformin use was associated with a lower rate of total exacerbations (aIRR 0.3; 95% CI 0.11, 0.77) and severe exacerbations (aIRR 0.29; 95% CI 0.1, 0.89). Among participants with COPD alone, metformin use was not associated with either outcome (Fig. 1). There was statistical evidence that the ACO modified the association of metformin use and rate of exacerbations (p-value for interaction < 0.05 for both comparisons).

These results were not qualitatively different in sensitivity analyses utilizing alternative definitions of ACO (Additional file 1: Figure S2). Replacement of comorbidity count with individual comorbidities did not qualitatively change results (not shown).

### Prevalent metformin use and cross-sectional secondary outcomes

Baseline cross-sectional associations between metformin use and SGRQ score, 6MWD, and FEV1pp are presented in Table 2. Among all participants, metformin use was associated with lower SGRQ score at baseline (adjusted mean difference [aMD] − 2.7; 95% CI − 5.3, − 0.2) which did not statistically differ between ACO and COPD (Table 2). Metformin use at baseline was not associated with differences in the rate of change for any secondary outcomes with no significant effect modification or subgroup effects by ACO status (Additional file 1: Table S2). A sensitivity analysis that included COPDGene participants who had not accrued follow-up time after the baseline visit revealed similar results (Additional file 1: Table S3).

**Table 2:** Adjusted mean difference (95% confidence interval) comparing metformin users to non-users for cross-sectional secondary outcomes

Outcome	All participants (n = 3969)	Subgroup analysis		
		Asthma-COPD overlap (n = 510)	COPD alone (n = 3459)	p-interaction
St. George respiratory questionnaire total score	**− 2.7 (− 5.3, − 0.2)**	-4 (-9.7, 1.7)	− 2.5 (− 5.3, 0.2)	0.6
Six-Minute Walk Distance (ft)	20 (− 29.7, 69.7)	− 20.8 (− 133.9, 92.3)	26.5 (− 26.7, 79.6)	0.4
Post-Bronchodilator FEV_1_ percent predicted	− 0.77 (− 3.9, 2.4)	− 3.6 (− 10.7, 3.5)	− 0.16 (− 3.5, 3.2)	0.4

Models are adjusted for age, sex, race, education, post-bronchodilator FEV_1_ percent predicted (except where it is the outcome), self-reported respiratory exacerbation in the prior 12 months (yes/no), smoking status, body mass index, inhaled corticosteroid use, oral corticosteroid use, other diabetes medication use, and comorbidity count. The p-value shown is for the interaction representing effect modification of Asthma-COPD overlap status on the association of metformin use with the outcome of interest. Lower St. George Respiratory Questionnaire score, higher six-minute walk distance, and higher FEV_1_ percent predicted favor metformin use. Bolded values are statistically significant at p < 0.05

### Metformin initiation and secondary outcomes

Among 2188 individuals not using metformin at baseline who attended the phase 2 follow-up, 108 reported metformin use at follow-up and thus were considered to have initiated metformin. Compared to those who did not use metformin, metformin initiation was associated with improved SGRQ score only in the group with ACO (aMD − 10.0; 95% CI − 18.7, − 1.2; p-interaction = 0.03). There were no associations between metformin initiation and 6MWD or FEV1pp or between metformin initiation and SGRQ score among participants with COPD alone (Table 3).

**Table 3:** Adjusted mean difference (95% confidence interval) in change of secondary outcomes from baseline to 5-year follow-up between metformin initiators and non-initiators

Outcome	All participants (n = 2188)	Subgroup analysis		
		Asthma-COPD overlap (n = 278)	COPD (n = 1910)	p-interaction
St. George respiratory questionnaire total score	− 1 (− 4.1, 2.1)	− **10 (**− **18.7, **− **1.2)**	0.27 (− 3, 3.6)	**0.03**
Six-Minute Walk Distance (ft)	5.2 (− 64, 74.4)	− 129.4 (− 337.7, 79)	23 (− 50.4, 96.5)	0.2
Post-Bronchodilator FEV_1_ percent predicted	1.4 (− 0.87, 3.7)	− 1.1 (− 7.7, 5.4)	1.8 (− 0.68, 4.3)	0.4

Models are adjusted for age, sex, race, education, post-bronchodilator FEV1pp (time-varying, excluded for FEV1pp outcome), smoking status (time-varying), body mass index (time varying), inhaled corticosteroid use (time-varying), oral corticosteroid use (time-varying), other diabetes medication use, comorbidity count. The p-value shown is for the three-way interaction between metformin use, time, and Asthma-COPD overlap status. Lower St. George Respiratory Questionnaire score, higher six-minute walk distance, and higher FEV_1_ percent predicted favor metformin use. Bolded values are statistically significant at p < 0.05

## Discussion

In this retrospective analysis of a large multi-center observational cohort study of COPD, metformin use was associated with a lower rate of respiratory exacerbations only among individuals with concurrent physician-diagnosed asthma. We further report that metformin initiation was associated with an improvement in quality of life preferentially among those with ACO. Taken together, these results suggest that metformin may have benefit in select individuals with COPD, presumably those that are driven by asthma-related pathophysiology. To our knowledge, this is the first report of this association.

Although there are no universally agreed-upon diagnostic criteria for ACO, the definition of asthma-COPD overlap used in this study is common and is the operative definition of ACO within COPDGene [10]. Specific treatment options for ACO are limited as major therapeutic trials of asthma and COPD commonly exclude participants with ACO [4]. Given individuals with ACO often report greater morbidity than those with COPD alone, increased attention to risk factors and treatment options for this condition is warranted.

Metformin is an oral medication used in the treatment of type 2 diabetes. Numerous effects have been described beyond improvements in insulin sensitivity and glucose tolerance [16], including amelioration of systemic and airway inflammation [17, 18]. Any potential mechanism of benefit for metformin in ACO is speculative. Metformin has been reported to inhibit airway smooth muscle (ASM) proliferation through AMPK-mediated inhibition of TGF-β1 signalling [19], a pathway that has also been demonstrated to impair ASM bronchorelaxation in response to beta-agonists [20]. Metformin decreased eosinophilic airway inflammation in an allergic asthma model [5] and an obese asthma model [23]. In the latter study, metformin treatment was also associated with an increase in regulatory T-lymphocytes, which have been shown in a separate study to reduce airway inflammation and hyperresponsiveness when administered intratracheally [22]. Studies of asthma using administrative data, which have excluded the presence of COPD, have reported that metformin use is associated with less exacerbations [6]. These translational and epidemiologic studies of metformin in asthma, combined with conflicting reports regarding metformin in COPD [8, 24], are consistent with our findings of possible benefit in ACO but not COPD. As metformin initiation was also associated with an improvement in SGRQ among participants with ACO, our report also suggests that this medication may play a role in attenuating both the daily impact of asthma on symptoms and quality of life and on more severe forms of disease exacerbation. Because associations were maintained with mutual adjustment for other diabetes medications, it is also plausible that any potential benefit may be specific to metformin rather than to general effects on glycemic control or insulin sensitivity.

Importantly, our results increase confidence that these findings are not driven by a healthy user effect, whereby users of preventative medications such as metformin are also more likely to adhere to chronic medications such as controllers for asthma or COPD [21]. The magnitude of such an effect would not be expected to be significantly different between individuals with ACO and COPD alone.

We additionally note that, although we did not find a statistically significant association between metformin use and respiratory outcomes among individuals with COPD, the size of our confidence intervals does not preclude the potential for clinically meaningful benefit or harm. The significance of a lower SGRQ score among all users of metformin, without consistent results in longitudinal analyses or the primary outcome, is unknown.

This study had several limitations. Our definition of ACO is based on participant report. Data necessary to test other definitions was not universally collected at the baseline visit, such as peripheral eosinophilia or serum markers of atopy. Because COPDGene did not comprehensively assess metabolic parameters at baseline, we were also unable to adjust for concurrent glycemic control. Thus, we were unable to explore whether metformin may improve asthma through effects on blood glucose independent of other aspects of metabolic function, although this may be less likely as associations were identified with concurrent adjustment for other diabetes medications that would also improve glycemic control. It is additionally possible that metformin use may represent diabetes and poor glucose control rather than medication use per se. However, the directionality of such an association would be expected to be in the opposite direction [25]. Additionally, medication use was not prospectively validated against medication lists or prescribing records, and dosages of medications were not collected, precluding assessment of dose–response relationships. Finally, with respect to the metformin initiator analysis, the exact time that metformin was started after the baseline visit was not collected, and thus some exposed person-time may be misclassified, though such misclassification would be expected to be conservative. The lack of information regarding time of metformin initiation also precluded investigation of exacerbation rate among metformin initiators.

## Conclusion

In conclusion, within a well-characterized observational cohort, we report a protective association between metformin use and respiratory outcomes preferentially in individuals with asthma-COPD overlap. These results support a potential role of metformin in select patients with concurrent COPD and asthma that warrants prospective investigation.

## Supplementary Information

**Additional file 1: Table S1.** Baseline characteristics of included participants by asthma-COPD overlap status (n=3969). **Table S2**. Adjusted mean difference (95% confidence interval) in change of secondary outcomes from baseline to 5-year follow-up between metformin users and non-users. **Table S3.** Adjusted mean difference (95% confidence interval) comparing metformin users to non-users for cross-sectional secondary outcomes including participants without follow-up data. **Figure S1.** STROBE diagram of cohort derivation. **Figure S2.** Incidence rate ratio (95% confidence interval) comparing metformin users to non-users for exacerbations stratified by alternative alternative asthma-COPD overlap (ACO) definitions (n=3969)

## Data Availability

Study data are available upon reasonable request through COPDGene at https://www.copdgene.org/.

## Abbreviations

6MWD: Six-minute walk distance
ACO: Asthma-COPD overlap
ASM: Airway smooth muscle
aIRR: Adjusted incidence rate ratio
aMD: Adjusted mean difference
BMI: Body mass index
COPD: Chronic obstructive pulmonary disease
COPDGene: The Genetic Epidemiology of COPD study
FEV_1_: Forced expiratory volume at 1 s
FEV_1_pp: Percent predicted forced expiratory volume at 1 s
MCID: Minimal clinically important difference
SD: Standard deviation
SGRQ: St. George Respiratory Questionnaire
