# Supplementary material for: Metformin use and respiratory outcomes in asthma-COPD overlap
Source: Respir Res. 2021 Feb 26;22:70. doi: 10.1186/s12931-021-01658-3 (PMC7908718; doi:10.1186/s12931-021-01658-3)
Supplement: Supplementary file 1 — Additional file 1: Table S1. Baseline characteristics of included participants by asthma-COPD overlap status (n=3969). Table S2. Adjusted mean difference (95% confidence interval) in change of secondary outcomes from baseline to 5-year follow-up between metformin users and non-users. Table S3. Adjusted mean difference (95% confidence interval) comparing metformin users to non-users for cross-sectional secondary outcomes including participants without follow-up data. Figure S1. STROBE diagram of cohort derivation. Figure S2. Incidence rate ratio (95% confidence interval) comparing metformin users to non-users for exacerbations stratified by alternative alternative asthma-COPD overlap (ACO) definitions (n=3969) [file 12931_2021_1658_MOESM1_ESM.docx]

**SUPPLEMENTARY MEDIA**
Metformin use and respiratory outcomes in asthma-COPD overlap

Tianshi David Wu, MD MHS; Ashraf Fawzy, MD MPH; Gregory L. Kinney, PhD MPH; Jessica Bon, MD,MS; Maniraj Neupane, MD, PhD; Vickram Tejwani, MD; Nadia N. Hansel, MD, MPH; Robert A. Wise, MD; Nirupama Putcha, MD, MHS; Meredith McCormack, MD MHS

Supplementary Tables

eTable 1. Baseline characteristics of included participants by asthma-COPD overlap status (n=3969)
eTable 2. Adjusted mean difference (95% confidence interval) in change of secondary outcomes from baseline to 5-year follow-up between metformin users and non-users
eTable 3. Adjusted mean difference (95% confidence interval) comparing metformin users to non-users for cross-sectional secondary outcomes including participants without follow-up data

Supplementary Figures

eFigure 1. STROBE diagram of cohort derivation
eFigure 2. Incidence rate ratio (95% confidence interval) comparing metformin users to non-users for exacerbations stratified by alternative alternative asthma-COPD overlap (ACO) definitions (n=3969)

eTable 1. Baseline characteristics of included participants by asthma-COPD overlap status (n=3969)

| Characteristic [N (%) or Mean±SD] | COPD Alone (n=3459) | Asthma-COPD Overlap (n=510) | p-value |
| --- | --- | --- | --- |
| Age | 64±8.3 | 60.5±8.7 | <0.01 |
| Female gender | 1522 (44) | 263 (52) | <0.01 |
| Black race | 622 (18) | 168 (33) | <0.01 |
| GOLD stage  GOLD 1  GOLD 2  GOLD 3  GOLD 4 | 630 (18)  1477 (43)  911 (26)  441 (13) | 54 (11)  232 (45)  144 (28)  80 (16) | <0.01 |
| Post-bronchodilator FEV1pp | 57.8±22.8 | 53.7±20.7 | <0.01 |
| Current smoker | 1372 (40) | 213 (42) | 0.4 |
| Body mass index  Underweight  Normal/overweight  Obese | 93 (3)  2300 (66)  1066 (31) | 11 (2)  302 (59.2)  197 (38.6) | <0.01 |
| Exacerbation in prior year | 1103 (32) | 260 (51) | <0.01 |
| Medications  Inhaled corticosteroids  Oral corticosteroids  Metformin  Insulin  Other diabetes medication | 342 (10)  134 (4)  202 (6)  20 (1)  163 (5) | 75 (15)  41 (9)  39 (8)  3 (1)  23 (5) | <0.01  <0.01  0.1  1  0.8 |
| Comorbidities  Comorbidity count  Coronary artery disease  Congestive heart failure  Stroke  Hypertension  High cholesterol | 2.4±1.8  330 (10)  135 (4)  117 (3)  1643 (48)  1488 (43) | 2.9±3.1  45 (9)  36 (7)  18 (4)  271 (53)  205 (40) | <0.01  0.6  <0.01  0.9  0.02  0.2 |

Asthma-COPD overlap defined as a doctor diagnosis of asthma before age 40. GOLD: Global Initiative for Chronic Obstructive Lung Disease; FEV1pp: Forced expired volume in one second, percent predicted

eTable 2. Adjusted mean difference (95% confidence interval) in change of secondary outcomes from baseline to 5-year follow-up between metformin users and non-users

|  |  | Subgroup Analysis | | |
| --- | --- | --- | --- | --- |
| Outcome | All participants (n=3969) | Asthma-COPD overlap (n=510) | COPD (n=3459) | p-interaction |
| St. George Respiratory Questionnaire Total Score | 0.89 (-1.6, 3.4) | 4.7 (-1.3, 10.7) | 0.33 (-2.4, 3.1) | 0.2 |
| Six-Minute Walk Distance (ft) | -41.6 (-100.2, 16.9) | -116.7 (-258.3, 24.8) | -28.2 (-90.4, 34.1) | 0.3 |
| Post-Bronchodilator FEV_1_ percent predicted | 1.4 (-0.9, 3.7) | 4 (-1.5, 9.5) | 0.84 (-1.9, 3.4) | 0.3 |

Models are adjusted for age, sex, race, education, post-bronchodilator FEV1pp (time-varying, excluded for FEV1pp outcome), smoking status (time-varying), body mass index (time varying), inhaled corticosteroid use (time-varying), oral corticosteroid use (time-varying), comorbidity count. The p-value shown is for the three-way interaction between metformin use, time, and Asthma-COPD overlap status. Lower St. George Respiratory Questionnaire score, higher six-minute walk distance, and higher FEV_1_ percent predicted favor metformin use.

eTable 3. Adjusted mean difference (95% confidence interval) comparing metformin users to non-users for cross-sectional secondary outcomes including participants without follow-up data

|  |  | Subgroup Analysis | | |
| --- | --- | --- | --- | --- |
| Outcome | All participants (n=4466) | Asthma-COPD overlap (n=603) | COPD (n=3863) | p-interaction |
| St. George Respiratory Questionnaire Total Score | -1.9 (-4.4, 0.52) | -3.5 (-9, 1.9) | -1.7 (-4.3, 0.97) | 0.5 |
| Six-Minute Walk Distance (ft) | 19.8 (-27.8, 67.4) | -1.4 (-1.9, 106) | 22.6 (-28.5, 73.7) | 0.7 |
| Post-Bronchodilator FEV_1_ percent predicted | -0.02 (-3, 2.9) | -1.3 (-7.9, 5.3) | 0.28 (-2.9, 3.5) | 0.7 |

Models are adjusted for age, sex, race, education, post-bronchodilator FEV1pp (time-varying, excluded for FEV1pp outcome), smoking status (time-varying), body mass index (time varying), inhaled corticosteroid use (time-varying), oral corticosteroid use (time-varying), comorbidity count. The p-value shown is for the three-way interaction between metformin use, time, and Asthma-COPD overlap status. Lower St. George Respiratory Questionnaire score, higher six-minute walk distance, and higher FEV_1_ percent predicted favor metformin use. Bolded values are statistically significant at p<0.05

eFigure 1. STROBE diagram of cohort derivation


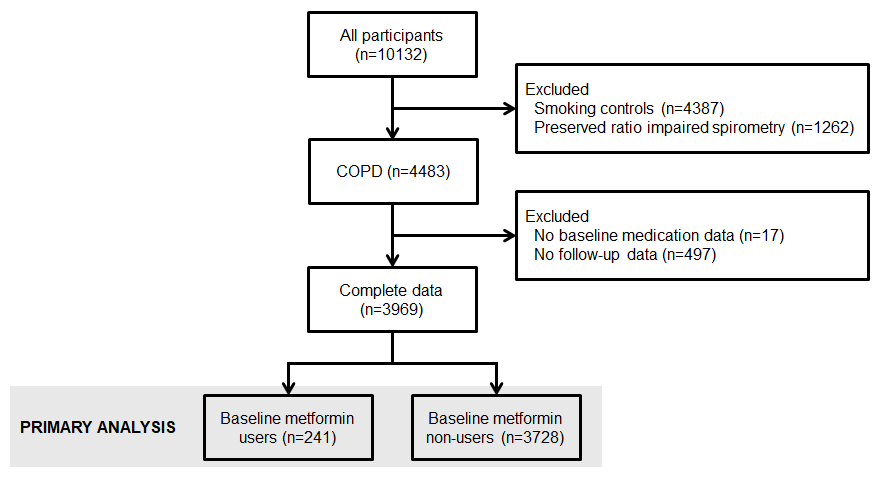


eFigure 2. Incidence rate ratio (95% confidence interval) comparing metformin users to non-users for exacerbations stratified by alternative alternative asthma-COPD overlap (ACO) definitions (n=3969)
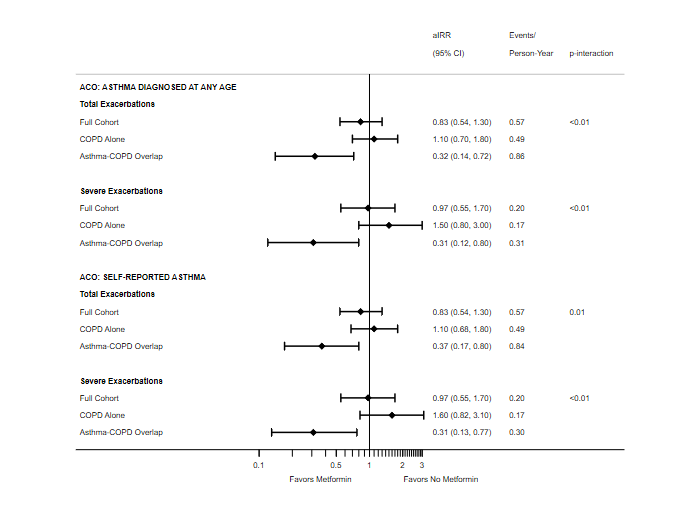

Results are adjusted for age, sex, race, education, post-bronchodilator FEV1pp, self-reported respiratory exacerbation in the prior 12 months (yes/no), smoking status, body mass index, inhaled corticosteroid use, oral corticosteroid use, other diabetes medication use, and comorbidity count. The p-value shown is for the interaction representing effect modification of Asthma-COPD overlap status on the association of metformin use with exacerbation rate.
